# Draft Genome Sequence of Laverivirus UC1, a Dicistrovirus-Like RNA Virus Featuring an Unusual Genome Organization

**DOI:** 10.1128/genomeA.00656-15

**Published:** 2015-07-02

**Authors:** Alexander L. Greninger, Joseph L. DeRisi

**Affiliations:** a Department of Biochemistry and Biophysics, UCSF, San Francisco, California, USA; b Howard Hughes Medical Institute, UCSF, San Francisco, California, USA

## Abstract

We report the draft genome sequence of Laverivirus UC1, assembled from San Francisco wastewater. This dicistronic RNA virus bears some similarity to dicistroviruses; however, it appears to have a unique genome organization relative to all other known RNA viruses.

## GENOME ANNOUNCEMENT

The picorna-like superfamily is a taxonomic unit of positive-stranded RNA viruses with conserved RNA-dependent RNA polymerase (RdRp), capsid, and helicase proteins that infect a broad range of hosts, including animals, plants, and insects (1–3). While performing weekly metagenomic sequencing of San Francisco wastewater, we assembled a contig of 8,440 nucleotides that yielded BLASTx alignment to the RNA-dependent RNA polymerase of the dicistrovirus *Solenopsis invicta* *virus-1* (30% amino acid identity), the ATPase/helicase of the dicistrovirus *Formica exsecta virus 1* (26% amino acid identity), and the capsid protein of the dicistrovirus aphid lethal paralysis virus (23% amino acid identity) (4, 5). The genome appears dicistronic encoding for two separate open reading frames (ORFs) in the standard genetic code of 2,094 and 5,604 nucleotides. However, unlike the dicistroviruses, the first ORF encodes for the RHV-like and CRPV-like capsid proteins, while the second ORF encodes for the ATPase/helicase and RNA-dependent RNA polymerase, similar to a dicistronic picorna-like superfamily organization. This differs from *Dicistroviridae* members that have the two ORFs in the opposite configuration (6). Noncoding regions include a 5′ untranslated region (UTR) of 493 nucleotides, an intergenic region of 162 nucleotides, and a 3′ UTR of 85 nucleotides.

We have given the name Laverivirus UC1 to this virus due to its discovery in wastewater and the Latin root “laver” for a water basin, as well as the lack of a known host. The host organisms of dicistroviruses are arthropods, though given the unique genome organization of Laverivirus this may not be the case. Of note, the plurality of nonchordate eukaryotic reads in the non-DNAsed sample aligned to the mountain pine beetle *Dendroctonus ponderosae* (7, 8). In the DNAsed sample, 3,512 reads were recovered to picornaviridae, including hits to human pathogens enterovirus, cardiovirus, parechovirus, Aichi virus, and salivirus (9–14).

This viral genome was recovered from a wastewater sample that was taken on 25 January 2010, one week after a large rainstorm. This same sample also contained novel ciliate and marine RNA viruses, along with a new picalivirus (15–18). Sample processing was performed on 1 liter of wastewater that was concentrated to <5 ml with particles between the sizes of 0.22 µm and 300 kDa using Millipore Pellicon XL 300-kDa filters and 0.22-micron spin columns. The sample was treated with micrococcal nuclease, and nucleic acid was extracted using a Zymo Viral DNA/RNA kit; half of the recovered nucleic acid was treated with DNase. The contig was discovered and assembled using PRICE version 1.0 and SURPI version 1.0 from a total of 15,719,690 paired-end 65-bp reads sequenced on an Illumina GAIIx split between these DNAsed and untreated nucleic acid preparations (19, 20).

### Nucleotide sequence accession number.

The GenBank accession number for Laverivirus UC1 is KF510029.
